# Pretreatment eosinophil counts as a predictive biomarker in non‐small cell lung cancer patients treated with immune checkpoint inhibitors

**DOI:** 10.1111/1759-7714.15100

**Published:** 2023-09-05

**Authors:** Eiji Takeuchi, Kensuke Kondo, Yoshio Okano, Seiya Ichihara, Michihiro Kunishige, Naoki Kadota, Hisanori Machida, Nobuo Hatakeyama, Keishi Naruse, Hirokazu Ogino, Hiroshi Nokihara, Tsutomu Shinohara, Yasuhiko Nishioka

**Affiliations:** ^1^ Department of Clinical Investigation National Hospital Organization Kochi Hospital Kochi Japan; ^2^ Department of Respiratory Medicine and Rheumatology, Graduate School of Biomedical Sciences Tokushima University Tokushima Japan; ^3^ Department of Respiratory Medicine National Hospital Organization Kochi Hospital Kochi Japan; ^4^ Department of Pathology National Hospital Organization Kochi Hospital Kochi Japan; ^5^ Department of Community Medicine for Respirology, Graduate School of Biomedical Sciences Tokushima University Tokushima Japan

**Keywords:** biomarker, immune checkpoint inhibitors, non‐small cell lung cancer, predictive factor, pretreatment eosinophils

## Abstract

**Background:**

The peripheral blood eosinophil count prior to treatment has potential as a predictive biomarker for a beneficial clinical response to cancer immunotherapies. Therefore, the present study investigated the impact of the eosinophil count on overall survival (OS) in non‐small cell lung cancer (NSCLC) patients treated with immune checkpoint inhibitors (ICI).

**Methods:**

We retrospectively reviewed all patients diagnosed with NSCLC and treated with ICI monotherapy between March 2016 and August 2021 at National Hospital Organization Kochi Hospital and Tokushima University.

**Results:**

A total of 166 patients were included. Fifty‐five patients had an eosinophil count of less than 100 cells/μL (Eo < 100). Nighty‐eight patients had an eosinophil count of 100 cells/μL or more, but less than 500 cells/μL (100 ≤ Eo < 500). Thirteen patients had an eosinophil count of 500 cells/μL or more (Eo ≥500). The median OS of all lung cancer patients was 476 days. The median OS of lung cancer patients with Eo <100, 100 ≤ Eo <500, and Eo ≥500 was 339, 667, and 143 days, respectively. A Kaplan–Meier univariate analysis showed a significant difference in OS between these three groups (*p* < 0.001). A Cox proportional regression analysis identified 100 ≤ Eo <500 (*p* = 0.04), ECOG PS score ≥ 2 (*p* = 0.02), tumor size ≥5 cm (*p* = 0.02), and PD‐L1 ≥ 1% (*p* = 0.01) as independent predictors of OS.

**Conclusion:**

OS was significantly longer in ICI‐treated NSCLC patients with a pretreatment eosinophil count of 100 ≤ Eo <500 than in the other patients and, thus, has potential as a new predictive biomarker.

## Graphical abstract

This report is the first to show that an optimal absolute eosinophil count before ICI treatment is a favorable predictive factor in ICI monotherapy for advanced and metastatic NSCLC patients. OS was significantly longer in ICI‐treated NSCLC patients with a pretreatment eosinophil count of 100 ≤ Eo <500 than in the other patients and thus has potential as a new predictive biomarker.

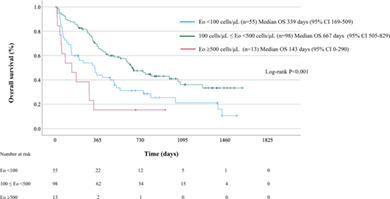

## INTRODUCTION

Immune checkpoint inhibitors (ICIs) have markedly changed the treatment of lung cancer. Nevertheless, not all patients benefit from ICIs, and even more severe side effects may occur. Therefore, the selection of suitable patients for ICIs is essential. Some clinically relevant prognostic and predictive markers have been reported in patients with non‐small cell lung cancer (NSCLC) treated with ICIs.^1^, ^2^ However, prognostic and predictive biomarkers beyond the expression status of programmed death ligand 1 (PD‐L1) are needed in daily clinical practice.

Eosinophils are effector cells in allergic diseases and parasitic infections and have diverse functions that differ from those of neutrophils and lymphocytes. Eosinophils are now recognized as a contributor to healthy homeostasis.^3^ Under normal conditions, eosinophil production is tightly regulated by the cytokine network.^4^ Tumor‐related eosinophilia may prolong the survival of some cancer patients.^5^, ^6^, ^7^ We previously reported a better prognosis for lung cancer patients with eosinophilic pleural effusion than for those with noneosinophilic effusion.^8^

The pretreatment peripheral blood eosinophil count in patients with melanoma has recently been suggested as a predictive marker for a beneficial clinical response to cancer immunotherapy.^9^, ^10^, ^11^, ^12^, ^13^ A previous study demonstrated that a low pretreatment eosinophil count was associated with poorer outcomes in patients with metastatic urothelial carcinoma treated with ICI.^14^ In patients with recurrent or metastatic squamous cell carcinoma of the head and neck, the eosinophil prognostic score has been useful as a novel prognostic score.^15^ In lung cancer patients, a baseline high absolute eosinophil count was associated with a better outcome to nivolumab treatment.^16^ Furthermore, lung cancer patients with a high baseline peripheral blood absolute eosinophil count had a higher objective response rate and longer median progression‐free survival and overall survival (OS).^17^, ^18^

On the other hand, the infiltration of tumor‐associated tissue by eosinophils has been identified as a poor prognostic factor in Hodgkin lymphoma.^19^ Eosinophils have been shown to play pleiotropic and opposing roles in the tumor microenvironment (TME).^20^, ^21^, ^22^ OS is the most robust indicator of cancer treatment outcomes. However, the relationship between the baseline peripheral blood eosinophil count and the prognosis of lung cancer patients treated with ICI has not yet been examined in detail.^23^ Therefore, we herein investigated the impact of the baseline peripheral blood eosinophil count on the OS of NSCLC patients treated with ICI.

## METHODS

### Patients

We retrospectively reviewed all patients diagnosed with advanced or metastatic NSCLC and treated with ICI monotherapy (nivolumab, pembrolizumab, and atezolizumab) between March 2016 and August 2021 at National Hospital Organization Kochi Hospital and Tokushima University.

### Data collection

We collected data on age, sex, smoking history, the Eastern Cooperative Oncology Group performance status (ECOG PS), baseline white blood cell count, neutrophil count, lymphocyte count, eosinophil count, neutrophil‐to‐lymphocyte ratio in serum (sNLR), C‐reactive protein (CRP), albumin (Alb), the histological type, the expression of PD‐L1, the genotypes of mutations, the type of ICI, line of ICI, date of ICI initiation, and the status of death. We also examined data obtained on the primary lesion size (maximum diameter measured on chest computed tomography), the number of metastatic sites (count of involved solid organs, not all sites), the status of specific metastasis (nonregional lymph nodes, contralateral lung, pleura, brain, liver, kidney, adrenal gland, and bone), and stage (according to the eighth edition of the tumor‐node‐metastasis classification of lung cancer). A computed tomography scan was performed for a radiological evaluation before ICI therapy. A radiographic complete response, partial response, stable disease, and progressive disease were defined according to the Response Evaluation Criteria in Solid Tumor, version 1.1. Objective response rates (RR) and disease control rates were described as a complete response plus a partial response and a complete response plus a partial response and stable disease, respectively.

### Statistical analysis

According to previous studies, the baseline median values of neutrophil and lymphocyte counts were selected as cutoff values.^24^ The baseline median values of CRP and Alb levels were also selected as cutoff values.^24^ NLR <5 and tumor size <5 cm were selected as cutoff values following previous studies.^25^, ^26^ Categorical and continuous variables are summarized using descriptive statistics. A one‐way ANOVA was used to examine differences between continuous variables. Pearson's chi‐squared test was used to test for relationships between categorical variables. OS was evaluated as the period from the day when ICI was initiated to the day of death from any cause using the Kaplan–Meier method. The Log‐rank test was performed to compare survival curves. A Cox proportional hazards model was used to estimate the hazard ratio (HR) with a 95% confidence interval (CI). We conducted all statistical analyses using SPSS statistics version 27.0 (IBM). The *p*‐values are presented without adjustments for multiple comparisons in an exploratory manner.

## RESULTS

### Patient characteristics

The present study included 166 advanced or metastatic NSCLC patients treated with ICI monotherapy. The clinical characteristics of enrolled patients are summarized in Table 1. The mean age of patients at ICI therapy was 69 years, 128 (77%) were male, and 130 (78%) were ex‐ or current smokers. Most patients (87%) had an ECOG PS of 0–1. Twenty‐one patients (13%) had postoperative recurrence, 31 (17%) were stage III, and 114 (69%) were stage IV. Ninety‐eight patients (59%) exhibited an adenocarcinoma histology, while 45 (27%) showed a squamous cell carcinoma histology. PD‐L1 expression was 1% or more in 132 patients (80%), but absent in 31 patients (19%). Six patients (4%) were harboring epidermal growth factor receptor (*EGFR*) mutations, two (1%) had the anaplastic lymphoma kinase (ALK) rearrangement, and one each (1%) harbored the rearranged during transfection (RET) and ROS proto‐oncogene 1 (ROS‐1) fusion. Forty‐five patients (27%) received ICIs as first‐line therapy. A total of 121 patients (73%) received ICIs as a second‐line or later treatment. Sixteen patients (10%) had liver metastasis. Forty‐two patients (25%) had brain metastasis. There were no patients with complications of atopic or allergic diseases. Seven patients were receiving baseline oral steroids beforehand.

**TABLE 1:** Baseline characteristics of the study population according to the absolute eosinophil count.

		Total	Eo <100	100 ≤ Eo <500	Eo ≥500	*p*‐value^a^
		*n* = 166	*n* = 55	*n* = 98	*n* = 13	
Age, years	Mean, (SD)	69 (9)	70 (9)	69 (9)	68 (9)	0.44
Sex, *n* (%)	Male	128 (77)	42 (76)	77 (79)	9 (69)	0.74
	Female	38 (23)	13 (24)	21 (21)	4 (31)	
Smoking history, *n* (%)	Yes	130 (78)	41 (75)	78 (80)	11 (85)	0.47
	No	30 (18)	13 (24)	15 (15)	2 (15)	
ECOG PS, *n* (%)	0–1	145 (87)	46 (84)	92 (94)	7 (54)	**<0.001**
	2–4	21 (13)	9 (16)	6 (6)	6 (46)	
Stage, *n* (%)	Recurrence	21 (13)	8 (15)	13 (13)	0 (0)	0.35
	III	31 (17)	9 (16)	21 (21)	1 (8)	
	IV	114 (69)	38 (69)	64 (65)	12 (92)	
Histological type, *n* (%)	Adeno	98 (59)	31 (56)	57 (58)	10 (77)	0.71
	Squamous	45 (27)	15 (27)	28 (29)	2 (15)	
	Others	23 (14)	9 (16)	13 (13)	1 (8)	
PD‐L1, *n* (%)	<1%	31 (19)	9 (16)	20 (20)	2 (15)	0.73
	≥1%	132 (80)	44 (80)	77 (79)	11 (84)	
	Missing	3 (2)	2 (4)	1 (1)	0 (0)	
Driver mutation, *n* (%)	None	151 (91)	50 (91)	90 (92)	11 (85)	0.90
	EGFR	6 (4)	3 (5)	2 (2)	1 (8)	
	ALK	2 (1)	1 (2)	1 (1)	0 (0)	
	RET	1 (1)	0 (0)	1 (1)	0 (0)	
	ROS‐1	1 (1)	0 (0)	1 (1)	0 (0)	
	Missing	5 (3)	1 (2)	3 (3)	1 (8)	
Treatment line, *n* (%)	1	45 (27)	11 (20)	30 (31)	4 (31)	0.35
	≥2	121 (73)	44 (80)	68 (69)	9 (69)	
ICI drug, *n* (%)	Pembrolizumab	130 (78)	42 (76)	77 (79)	11 (84)	0.88
	Nivolumab	35 (21)	13 (24)	20 (20)	2 (15)	
	Atezolizumab	1 (1)	0 (0)	1 (1)	0 (0)	
Liver metastasis, *n* (%)	No	150 (90)	48 (87)	89 (91)	13 (100)	0.20
	Yes	16 (10)	7 (12)	9 (9)	0 (0)	
Brain metastasis, *n* (%)	No	124 (75)	39 (71)	75 (77)	10 (77)	0.73
	Yes	42 (25)	16 (29)	23 (23)	3 (23)	
Tumor size, mm	Mean, (SD)	44 (24)	48 (23)	39 (22)	56 (32)	**0.009**
White blood count/μL	Mean, (SD)	7229 (3404)	6944 (3245)	7100 (3427)	9407 (3362)	0.053
Neutrophils, %	Mean, (SD)	67 (10)	68 (10)	66 (9)	67 (11)	0.45
Lymphocytes, %	Mean, (SD)	22 (9)	22 (9)	22 (9)	16 (9)	**0.047**
sNLR, ratio	Mean, (SD)	3.9 (2.5)	3.7 (1.9)	3.8 (2.6)	5.6 (3.1)	**0.04**
CRP, mg/dL	Mean, (SD)	2.6 (3.4)	2.5 (3.6)	2.4 (3.3)	3.8 (3.4)	0.40
Alb, g/dL	Mean, (SD)	3.5 (0.7)	3.4 (0.6)	3.5 (0.6)	3.0 (0.6)	**0.04**

*Note*: The *p*‐values <0.05 are in bold.

Abbreviations: 100 ≤ Eo <500, 100 cells/μL ≤eosinophils <500 cells/μL; Alb, albumin; ALK, anaplastic lymphoma kinase; CRP, C‐reactive protein; ECOG PS, Eastern Cooperative Oncology Group Performance Status; EGFR, epidermal growth factor receptor; Eo <100, eosinophils <100 cells/μL; Eo ≥500, eosinophils ≥500 cells/μL; ICI, immune checkpoint inhibitor; PD‐L1, programmed death ligand 1; RET, rearrangement during transfection; ROS1, ROS proto‐oncogene 1; sNLR, neutrophil‐to‐lymphocyte ratio in serum.

^a^ *p*‐value: A one‐way ANOVA for continuous comparisons and the chi‐squared test for categorical comparisons.

Patients in the present study were divided into three groups based on previously reported absolute eosinophil counts.^4^, ^14^ As a low pretreatment eosinophil count of less than 100 cells/μL was associated with poorer outcomes in patients with metastatic urothelial carcinoma treated with ICI,^14^ we selected a lower cutoff level of 100 cells/μL. The upper limit for a normal eosinophil count is 500 cells/μL.^4^ Eosinophils are now recognized as a contributor to healthy homeostasis.^3^ Under normal conditions, eosinophil production is closely regulated by the cytokine network. Therefore, an upper cutoff level of 500 cells/μL was used in the present study. The mean age of 55 patients with an eosinophil count of less than 100 cells/μL (Eo <100) was 70 years, 42 (76%) were male, and 41 (75%) were ex‐ or current smokers. Forty‐six patients (84%) had an ECOG PS of 0–1. Eight patients (15%) had postoperative recurrence, nine (16%) had stage III, and 38 (69%) had stage IV. Thirty‐one patients (56%) showed an adenocarcinoma histology, while 15 (27%) exhibited a squamous cell carcinoma histology. PD‐L1 expression was 1% or more in 44 patients (80%), but was absent in nine patients (16%). Three patients (5%) harbored *EGFR* mutations, and one (2%) had the ALK rearrangement. Eleven patients (20%) received ICIs as first‐line therapy. Forty‐four patients (80%) received ICIs as a second‐line or later treatment. Seven (12%) had liver metastasis. Sixteen patients (29%) had brain metastasis. Five patients were receiving baseline oral steroids beforehand.

The mean age of 98 patients with an eosinophil count of 100 cells/μL or more, but less than 500 cells/μL (100 ≤ Eo <500) was 69 years; 77 (79%) were male, and 78 (80%) were ex‐ or current smokers. Ninety‐two patients (94%) had an ECOG PS of 0–1. Thirteen patients (13%) had postoperative recurrence, 21 (21%) had stage III, and 64 (65%) had stage IV. Fifty‐seven patients (58%) exhibited an adenocarcinoma histology, while 28 (29%) showed a squamous cell carcinoma histology. PD‐L1 expression was 1% or more in 77 patients (79%), but was absent in 20 patients (20%). Two patients (2%) harbored *EGFR* mutations, one (1%) had the ALK rearrangement and one each (1%) harbored the RET and ROS‐1 fusion. Thirty patients (31%) received ICIs as first‐line therapy. Sixty‐eight patients (69%) received ICIs as a second‐line or later treatment. Nine (9%) had liver metastasis. Twenty‐three patients (23%) had brain metastasis. Two patients were receiving baseline oral steroids beforehand.

The mean age of 13 patients with an eosinophil count of 500 cells/μL or more (Eo ≥500) was 68 years, nine (69%) were male, and 11 (85%) were ex‐ or current smokers. Seven patients (54%) had an ECOG PS of 0–1. One patient (8%) had stage III and 12 (92%) had stage IV. Ten patients (77%) exhibited an adenocarcinoma histology, while two (15%) showed a squamous cell carcinoma histology. PD‐L1 expression was 1% or more in 11 patients (84%), but absent in two patients (15%). One patient (8%) harbored *EGFR* mutation. Four patients (31%) received ICIs as first‐line therapy. Nine patients (69%) received ICIs as a second‐line or later treatment. None (0%) had liver metastasis. Three patients (23%) had brain metastasis. No patients were receiving baseline oral steroids beforehand.

Significant differences were observed in ECOG PS, tumor size, lymphocytes, sNLR, and Alb between the three groups.

### OS of NSCLC patients treated with ICI

**FIGURE 1:**
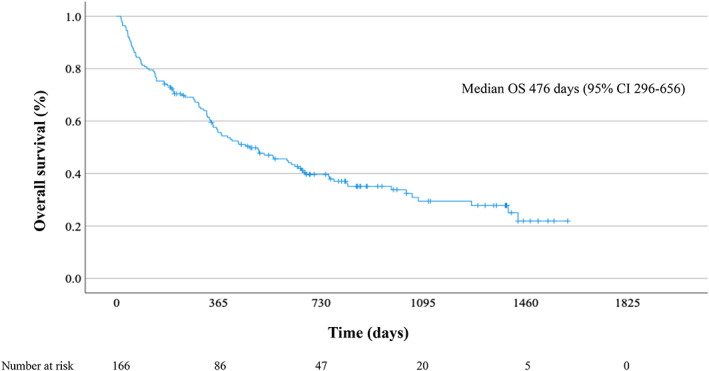
Overall survival of non‐small cell lung cancer patients treated with immune checkpoint inhibitors.

The median OS of all 166 NSCLC patients treated with ICIs was 476 days (95% CI: 296–656) (Figure 1).

### RR and disease control rates according to the absolute eosinophil count

**FIGURE 2:**
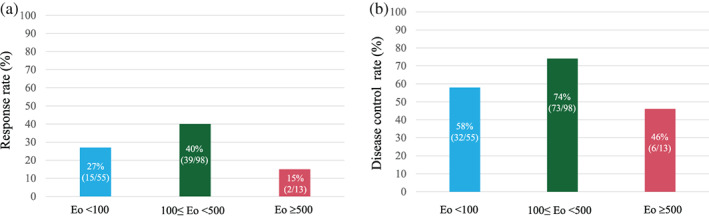
Response rates (a) and disease control rates (b) according to pretreatment absolute eosinophil counts (Eo).

The RR of ICI‐treated NSCLC patients with Eo <100 (*n* = 55), 100 ≤ Eo <500 (*n* = 98), and Eo ≥500 (*n* = 13) were 27% (95% CI: 16%–41%), 40% (95% CI: 30%–50%), and 15% (95% CI: 1.9%–45%), respectively (Figure 2a). The disease control rates of ICI‐treated NSCLC patients with Eo <100 (*n* = 55), 100 ≤ Eo <500 (*n* = 98), and Eo ≥500 (*n* = 13) were 58% (95% CI: 44%–71%), 74% (95% CI: 65%–83%), and 46% (95% CI: 19%–75%), respectively (Figure 2b).

### OS of NSCLC patients treated with ICI according to the absolute eosinophil count

**FIGURE 3:**
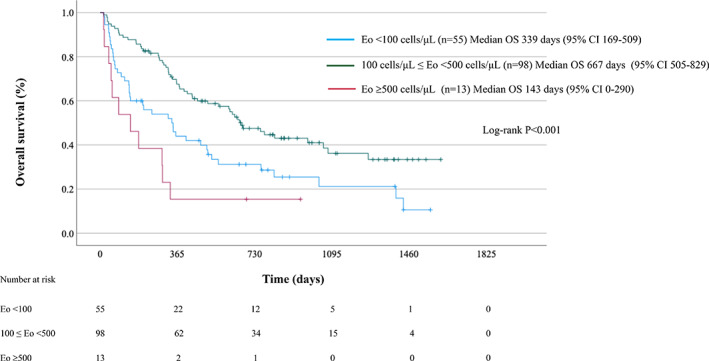
Patient survival according to pretreatment absolute eosinophil counts (Eo).

The median OS of ICI‐treated NSCLC patients with Eo <100 (*n* = 55), 100 ≤ Eo <500 (*n* = 98), and Eo ≥500 (*n* = 13) were 339 days (95% CI: 169–509), 667 days (95% CI: 505–829), and 143 days (95% CI: 0–290), respectively (Figure 3). A significant difference was observed in OS between the three groups (*p* < 0.001).

### Univariate analysis

As shown in Table 2, in univariate Cox proportional regression analyses, ECOG PS score ≥ 2 (HR, 2.65; 95% CI: 1.57–4.48; *p* < 0.001), liver metastasis (HR, 1.87; 95% CI: 1.04–3.35; *p* = 0.04), 100 ≤ Eo <500 (HR, 0.3; 95% CI: 0.15–0.57; *p* < 0.001), neutrophils ≥69% (HR, 1.66; 95% CI: 1.14–2.43; *p* = 0.009), lymphocytes ≥22% (HR, 0.61; 95% CI: 0.41–0.89; *p* = 0.01), CRP ≥1 mg/dL (HR, 1.74; 95% CI: 1.18–2.55; *p* = 0.005), Alb ≥3.5 g/dL (HR, 0.61; 95% CI: 0.41–0.90; *p* = 0.01), and tumor size ≥5 cm (HR, 1.96; 95% CI: 1.34–2.87; *p* < 0.001) were identified as significant factors for OS during ICI monotherapy, whereas age, sex, stage, histological type, brain metastasis, sNLR, and PD‐L1 ≥ 1% were not.

**TABLE 2:** Univariate Cox regression analysis of clinical and laboratory parameters associated with the overall survival of non‐small cell lung cancer patients treated with immune checkpoint inhibitors.

Parameters	Category	Hazard ratios	95% CI of HR	*p*‐value
ECOG PS	2 ~ 4	2.65	1.57–4.48	**<0.001**
	0 ~ 1	Reference		
Age	≥75	1.02	0.66–1.61	0.9
	<75	Reference		
Sex	Female	1.26	0.81–1.95	0.3
	Male	Reference		
Stage	recurrence	0.61	0.32–1.14	0.12
	III	0.68	0.40–1.15	0.15
	IV	Reference		
Histological type	Sq	1.19	0.79–1.79	0.4
	Non‐Sq	Reference		
Liver metastasis	Yes	1.87	1.04–3.35	**0.04**
	No	Reference		
Brain metastasis	Yes	1.19	0.77–1.85	0.4
	No	Reference		
Eosinophils	<100/μL	0.55	0.28–1.07	0.07
	100/μL≤, <500/μL	0.30	0.15–0.57	**<0.001**
	≥500/μL	Reference		
Neutrophils	≥69%	1.66	1.14–2.43	**0.009**
	<69%	Reference		
Lymphocytes	≥22%	0.61	0.41–0.89	**0.01**
	<22%	Reference		
sNLR, ratio	<5	0.75	0.47–1.18	0.21
	≥5	Reference		
CRP	≥1 mg/dL	1.74	1.18–2.55	**0.005**
	<1 mg/dL	Reference		
Alb	≥3.5 g/dL	0.61	0.41–0.90	**0.01**
	<3.5 g/dL	Reference		
Tumor size	≥5 cm	1.96	1.34–2.87	**<0.001**
	<5 cm	Reference		
PD‐L1	≥1%	0.77	0.49–1.23	0.27
	<1%	Reference		

*Note*: The *p*‐values <0.05 are in bold.

Abbreviations: Alb, albumin; CI, confidence interval; CRP, C‐reactive protein; ECOG PS, Eastern Cooperative Oncology Group Performance Status; HR, hazard ratio; Non‐Sq, nonsquamous cell carcinoma; PD‐L1, programmed death ligand 1; sNLR, neutrophil‐to‐lymphocyte ratio in serum; Sq, squamous cell carcinoma.

### Multivariate analysis

Variables with *p*‐values ≤0.2 in univariate models and clinically more important PD‐L1 were analyzed in multivariate models. In the Cox proportional regression analysis, ECOG PS score ≥ 2 (HR, 2.14; 95% CI: 1.16–3.96; *p* = 0.02), 100 ≤ Eo <500 (HR, 0.39; 95% CI: 0.16–0.94; *p* = 0.04), tumor size ≥5 cm (HR, 1.79; 95% CI: 1.08–2.96; *p* = 0.02), and PD‐L1 ≥ 1% (HR, 0.51; 95% CI: 0.31–0.85; *p* = 0.01) correlated with OS (Table 3), whereas stage, liver metastasis, neutrophils, lymphocytes, CRP, and Alb did not.

**TABLE 3:** . Multivariate Cox regression analysis of clinical and laboratory parameters associated with the overall survival of non‐small cell lung cancer patients treated with immune checkpoint inhibitors.

Parameters	Category	Hazard ratios	95% CI of HR	*p*‐value
ECOG PS	2 ~ 4	2.14	1.16–3.96	**0.02**
	0 ~ 1	Reference		
Stage	Recurrence	0.72	0.36–1.44	0.3
	III	0.78	0.42–1.43	0.4
	IV	Reference		
Liver metastasis	Yes	1.32	0.67–2.57	0.4
	No	Reference		
Eosinophils	<100/μL	0.64	0.25–1.61	0.3
	100/μL≤, <500/μL	0.39	0.16–0.94	**0.04**
	≥500/μL	Reference		
Neutrophils	≥69%	1.51	0.67–3.42	0.3
	<69%	Reference		
Lymphocytes	≥22%	1.09	0.49–2.43	0.8
	<22%	Reference		
CRP	≥1 mg/dL	1.11	0.63–1.95	0.7
	<1 mg/dL	Reference		
Alb	≥3.5 g/dL	1.16	0.68–1.95	0.6
	<3.5 g/dL	Reference		
Tumor size	≥5 cm	1.79	1.08–2.96	**0.02**
	<5 cm	Reference		
PD‐L1	≥1%	0.51	0.31–0.85	**0.01**
	<1%	Reference		

*Note*: The *p*‐values <0.05 are in bold.

Abbreviations: Alb, albumin; CI, confidence interval; CRP, C‐reactive protein; ECOG PS, Eastern Cooperative Oncology Group Performance Status; HR, hazard ratio; PD‐L1, programmed death ligand 1.

## DISCUSSION

The present results demonstrated that OS was longer in ICI‐treated NSCLC patients with a pretreatment eosinophil count of 100 ≤ Eo <500 than in the other patients. This is the first study to show that an optimal baseline peripheral blood eosinophil count may be an excellent predictive factor for lung cancer patients treated with ICIs.

Eosinophils are effector cells in allergic diseases and parasitic infections and have diverse functions that differ from those of neutrophils and lymphocytes. Eosinophils are now recognized as a contributor to healthy homeostasis.^3^ Under normal conditions, eosinophil production is tightly regulated by the cytokine network. The normal eosinophil count in peripheral blood ranges between 50 and 500 cells/μL.^4^

Tumor‐related eosinophilia may prolong the survival time of some cancer patients.^5^, ^6^, ^7^ We recently demonstrated that the prognosis of lung cancer patients with eosinophilic pleural effusion was better than that of patients with noneosinophilic effusion.^8^ Similar findings have been reported for oral squamous epithelial cancer, nasopharyngeal cancer,^27^ esophageal cancer,^28^ lung cancer,^29^ colorectal cancer,^30^, ^31^ prostate cancer,^32^ and penis cancer.^33^ The prognosis of patients is good if the infiltration of eosinophils into tumor tissue and the degranulation of eosinophils in tumor tissue are both observed. On the other hand, the infiltration of tumor‐associated tissue by eosinophils is a poor prognostic factor in Hodgkin lymphoma.^19^ In a knockout model, the infiltration of tumor‐related tissues by eosinophils was identified as a risk factor for oral cancer.^34^ Eosinophils have been reported to play pleiotropic and opposing roles in the TME.^20^, ^21^, ^22^

The peripheral blood eosinophil count in patients with melanoma before treatment has recently been suggested as a predictive marker for a beneficial clinical response to cancer immunotherapy^9^, ^10^, ^11^, ^12^, ^13^ A low pretreatment eosinophil count has been associated with poorer outcomes in patients with metastatic urothelial carcinoma treated with ICIs.^14^ In patients with recurrent or metastatic squamous cell carcinoma of the head and neck, the eosinophil prognostic score has been useful as a novel prognostic score.^15^ Eosinophil and leukocyte counts predicted progression‐free survival in relapsed or refractory classical Hodgkin lymphoma patients treated with ICIs.^35^

In lung cancer patients, a baseline high absolute eosinophil count (Eo ≥ 150 cells/μL) was associated with a better outcome to nivolumab treatment.^16^ Furthermore, lung cancer patients with a high baseline peripheral blood absolute eosinophil count (Eo ≥ 125 cells/μL) had a higher objective response rate and longer median progression‐free survival.^17^ A baseline high absolute eosinophil count (Eo ≥ 130 cells/μL) was a predictive biomarker of clinical benefits and immune‐related adverse events in NSCLC patients treated with ICIs.^18^ OS is the most robust indicator of cancer treatment outcomes. However, the relationship between the baseline peripheral blood eosinophil count and the prognosis of lung cancer patients treated with ICIs has not yet been examined in detail.^23^ In the present study, OS was significantly longer in patients with 100 ≤ Eo <500 than in the other patients treated with ICIs (Figure 2). Similar results were obtained when patients receiving oral steroids were excluded.

Many clinically relevant prognostic and predictive markers have been reported in NSCLC cancer patients treated with ICIs.^1^, ^2^ Laboratory, clinical, and genetic markers are prognostic and predictive factors.^1^ In the present study, the multivariable analysis identified 100 ≤ Eo <500 (*p* = 0.04), ECOG PS score ≥ 2 (*p* = 0.02), tumor size ≥5 cm (*p* = 0.02), and PD‐L1 ≥ 1% (*p* = 0.01) as independent predictors of OS. These results, except for eosinophils, are consistent with previous findings. PD‐L1 was not identified as a predictive factor in the univariate analysis, but was in the multivariate analysis. Some confounding factors not identified in the univariate analysis as predictors of OS may be significant after adjustments.

In the present study, patients with Eo ≥500 had a worse prognosis; however, the reason for this currently remains unclear. Nevertheless, eosinophils are now recognized as a contributor to healthy homeostasis.^3^ The eosinophil count is associated with malignant hematological diseases and all‐cause mortality. These relationships are U‐shaped.^36^ Eosinophils were shown to recruit regulatory T cells via the production of C‐C motif chemokine 22, which facilitated pulmonary metastasis in mice.^37^ Furthermore, eosinophils produce interleukin (IL)‐13, which polarizes macrophages towards the M2‐like immunosuppressive phenotype.^38^ Eosinophils have been shown to produce many growth factors that directly affect tumor growth, metastatic spread, matrix remodeling, and tumor‐associated blood vessels.^20^ Eosinophils have also been reported to play pleiotropic and opposing roles in the TME.^20^, ^21^, ^22^ Phenotypic studies on eosinophils in asthma mouse models revealed eosinophils with different locations, morphologies, and gene and cytokine expression profiles, reflecting other functions.^39^ A previous study proposed the classification of eosinophils into different phenotypes.^3^ Therefore, functional studies on eosinophils in humans are warranted. An optimal baseline eosinophil count may be necessary for ICI therapy to exert its antitumor effects.

In humans, immunotherapy with IL‐2,^40^, ^41^ IL‐4,^42^ granulocyte‐macrophage colony‐stimulating factor,^43^ or tumor vaccines often results in peripheral blood eosinophilia.^44^ The intrapleural administration of IL‐2 has been shown to induce significant eosinophilic pleural effusion.^45^ Eosinophils infiltrate a tumor and their subsequent activation promotes the infiltration of T cells into the tumor.^6^, ^46^ Activated eosinophils have also been reported to promote tumor‐specific CD8^+^ T cell infiltration and tumor rejection and prolong survival by improving TME.^46^ Eosinophils activated by IL‐33 induced the recruitment and activation of CD8^+^ T cells and natural killer cells in melanoma‐bearing mice.^47^ In patients with melanoma, eosinophils activated by ICI therapy may contribute to the migration of CD8^+^ T cells to tumor sites.^12^ A previous study reported that tumor‐infiltrating eosinophils consisted of degranulating eosinophils and were essential for tumor rejection independently of CD8^+^ T cells in the colorectal cancer model.^48^ Therefore, eosinophils may play a crucial role in cancer immunotherapy.

The limitations of the present study need to be addressed. This was a two‐center retrospective analysis conducted with heterogeneous data from patient cohorts and, as such, the results obtained are speculative and not definitive. Furthermore, multicenter studies are needed due to the small number of cases. We reported early mortality factors in ICI monotherapy for advanced or metastatic NSCLC.^24^ The same population was used in the present study and our previous research.^24^ However, the previous study identified early mortality factors during ICI treatment. The present study focused on the impact of the eosinophil count on the long‐term prognosis of ICI treatment. These two studies investigated entirely different and essential issues. Although we need to consider these limitations when interpreting the present results, this study is of value because an optimal absolute eosinophil count before ICI treatment was herein confirmed for the first time as a favorable predictive factor in ICI monotherapy for advanced and metastatic NSCLC. Simple clinical parameters may easily predict the prognosis of patients treated with a single‐agent ICI against advanced or metastatic NSCLC and are clinically useful. The role of eosinophils in cancer immunotherapy has not yet been elucidated in detail. However, eosinophils may be essential accessory cells for cancer immunotherapy. Therefore, a more detailed understanding of the relationship between eosinophils and cancer immunotherapy is needed, and further advances in basic and clinical cancer research in this area are required.

In conclusion, we demonstrated that OS was significantly longer in ICI‐treated NSCLC patients with a pretreatment eosinophil count of 100 ≤ Eo <500 than in the other patients and may represent a new predictive biomarker.

## AUTHOR CONTRIBUTIONS

Conceptualization and design: Eiji Takeuchi, Hiroshi Nokihara; Data collection and analysis: Kensuke Kondo, Yoshio Okano, Seiya Ichihara, Michihiro Kunishige, Naoki Kadota, Hisanori Machida, Nobuo Hatakeyama, Keishi Naruse, Hirokazu Ogino, Tsutomu Shinohara; Formal analysis and investigation: Eiji Takeuchi; Writing–original draft preparation: Eiji Takeuchi; Writing–review and editing: Hirokazu Ogino, Hiroshi Nokihara, Tsutomu Shinohara, Yasuhiko Nishioka; Supervision: Hiroshi Nokihara, Tsutomu Shinohara, Yasuhiko Nishioka. All authors read and approved the final manuscript.

## FUNDING INFORMATION

There is no funding to report.

## CONFLICT OF INTEREST STATEMENT

The authors declare that they have no conflicts of interest.
